# The prevalence of perceived stigma and self-blame and their associations with depression, emotional well-being and social well-being among advanced cancer patients: evidence from the APPROACH cross-sectional study in Vietnam

**DOI:** 10.1186/s12904-021-00803-5

**Published:** 2021-07-07

**Authors:** Nguyen Tuong Pham, Jia Jia Lee, Nhu Hiep Pham, Thi Do Quyen Phan, Khoa Tran, Hoai Bao Dang, Irene Teo, Chetna Malhotra, Eric A. Finkelstein, Semra Ozdemir

**Affiliations:** 1 Oncology Center, Hue Central Hospital, 16 Lê Lợi, Hue City, Vĩnh Ninh Vietnam; 2 Lien Centre for Palliative Care, Duke-NUS Medical School, 8 College Road, Singapore, 169857 Singapore; 3 Signature Programme in Health Services and System Research, Duke-NUS Medical School, 8 College Road, Singapore, 169857 Singapore

**Keywords:** Perceived stigma, Behavioural self-blame, Characterological self-blame, Vietnam, Advanced cancer

## Abstract

**Background:**

There is very limited evidence on the existence of cancer-related perceived stigma and self-blame among patients with advanced cancer in Asia, and how they are associated with psychosocial outcomes. This study aimed to address the gap in the current literature by (1) assessing perceived stigma, behavioural self-blame and characterological self-blame among Vietnamese patients with advanced cancer, and (2) investigating the associations of perceived stigma and self-blame (behavioural and characterological) with depression, emotional well-being and social well-being.

**Methods:**

This cross-sectional study involved 200 Vietnamese patients with stage IV solid cancer. Depression was measured using the Center for Epidemiologic Studies Depression (CES-D) Scale. Emotional well-being and social well-being were measured with the relevant domains of the Functional Assessment of Cancer Therapy-General (FACT-G) scale. Perceived stigma was assessed using the sense of stigma subscale of Kissane’s Shame and Stigma Scale. Behavioural self-blame and characterological self-blame were measured by the patients’ answers to the questions on whether their cancer was due to patient’s behaviour or character. Multivariable linear regressions were used to investigate the associations while controlling for patient characteristics.

**Results:**

Approximately three-fourths (79.0%, *n* = 158) of the participants reported perceived stigma with an average score of 20.5 ± 18.0 (out of 100). More than half of the participants reported behavioural self-blame (56.3%, *n* = 112) or characterological self-blame (62.3%, *n* = 124). Higher perceived stigma was associated with lower emotional well-being (ß = -0.0; *p* = 0.024). Behavioural self-blame was not significantly associated with depressive symptoms, emotional well-being or social well-being. Patients who reported characterological self-blame reported greater depressive symptoms (ß = 3.0; *p* = 0.020) and lower emotional well-being (ß = -1.6; *p* = 0.038).

**Conclusion:**

Perceived stigma and self-blame were common amongst Vietnamese advanced cancer patients. Perceived stigma was associated with lower emotional well-being while characterological self-blame were associated with greater depressive symptoms and lower emotional well-being. Interventions should address perceived stigma and self-blame among this population.

**Supplementary Information:**

The online version contains supplementary material available at 10.1186/s12904-021-00803-5.

## Background

The perception of stigma and self-blame among cancer patients is well-documented in several countries [1–9]. Perceived stigma in relation to being a cancer patient has been observed across all cancer types [1, 6, 10–12], and refers to the patient’s perception that others feel prejudice against them because of their cancer diagnosis which results in less social acceptance [12, 13]. Self-blame refers to the attribution of self as the cause of a situation, and can be either behavioural or characterological depending on the focus of blame [14]. Behavioural self-blame in the context of cancer focuses on one’s own risky health behaviour which is modifiable, such as smoking and drinking. On the contrary, characterological self-blame focuses on an individual’s character that is relatively hard to modify, such as the type of person they are.

Understanding cancer patients’ perceptions of being stigmatized and self-blame is important as these are parts of a patient’s experience with the disease and may lead to additional burden on cancer patients, further reducing their quality of life [6, 7, 9]. Several studies have shown that perceived stigma is associated with higher depressive symptoms, and lower emotional and social well-being amongst cancer patients [1, 6, 10–12]. Evidence on the associations of self-blame with depression, emotional well-being and social well-being, however, are limited and not conclusive [6, 11, 15–18]. In addition, evidence on self-blame seems to suggest that characterological self-blame was more detrimental on psychological well-being than behavioural self-blame [19, 20]. The majority of the studies mentioned above, however, were conducted in Western countries. Culture might play a crucial role in influencing how individuals perceive and respond to life-threatening diseases [21]. Beliefs such as cancer being contagious or a punishment from God still exist amongst people of Asian origins [21–23]. In addition, avoidance of cancer-related communication is very common in Asian countries [24, 25]. Such differences in cultural belief and practice may affect aspects of the experience of Asian patients, such as cancer-related stigma and self-blame, differently than those of Western societies. However, very little is known about perceived stigma and self-blame among cancer patients in Asian countries like Vietnam and the extent to which they are associated with psychosocial well-being. In Vietnam, the burden of cancer has risen rapidly in recent decades. An estimation by the International Agency for Research on Cancer (IARC) reported a total of 164,671 new cases and 114,871 cancer deaths in Vietnam in 2018 [26, 27]. These figures are three times higher than in 1990 [28]. Despite the drastically increasing cancer burden in Vietnam over the past thirty years, studies focusing on the underlying factors for psychosocial well-being of cancer patients in Vietnam remain scarce.

The aims of this study were 1) to assess the prevalence of perceived stigma and self-blame among advanced cancer patients in Vietnam, and 2) to investigate the associations of perceived stigma and self-blame (behavioural and characterological) with psychosocial well-being. We hypothesized that patients who reported perceived stigma, behavioural or characterological self-blame will report higher depressive symptoms, lower emotional well-being, and lower social well-being. Our findings will serve as an initial situational analysis for policy makers and health care providers to understand the relationship between perceived stigma, self-blame and psychosocial well-being amongst advanced cancer patients in Vietnam.

## Methods

### Participants and study setting

This study analysed data collected from the Vietnam site of the Asian Patient Perspectives Regarding Oncology Awareness, Care and Health (APPROACH) study, a cross-sectional multi-country study [29]. Data was collected by trained interviewers using a structured questionnaire through face-to-face interviews with inpatient and outpatient participants recruited at the Hue Central Hospital in Vietnam. The inclusion criteria included (1) being at least 21 years of age; (2) having been diagnosed with stage 4 solid cancer; (3) being aware of cancer diagnosis; (4) being a citizen of Vietnam. Ethics approvals were obtained from the National University of Singapore-Institutional Review Board (NUS-IRB B-15–319), Singapore and Hue Central Hospital Ethics Committee, Vietnam (230/QD-BVH-HDDD).

**Fig. 1:**
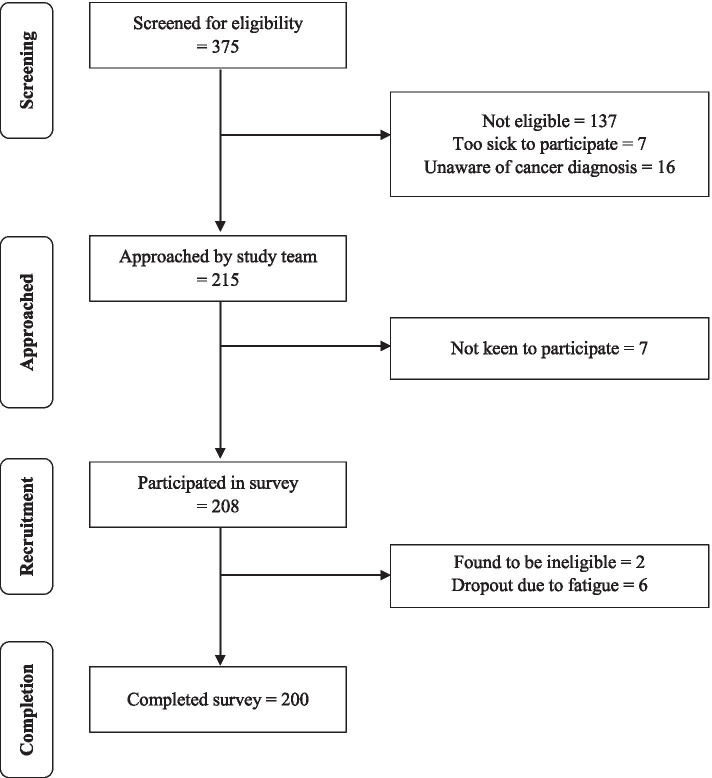
Recruitment flowchart

Recruitment was carried out between February 2018 to July 2018 (Fig. 1). The medical records of 375 patients were screened for eligibility. During the initial screening, a total of 238 patients were found to be eligible. Of these, 16 were not aware of their cancer diagnosis and therefore were not recruited. From the remaining participants, 7 were too ill and 7 were not interested in the study. Informed consent was then obtained from the remaining 208 participants. Of the 208 participants, 2 were subsequently found to be ineligible while 6 participants decided to withdraw from the study due to fatigue. One participant did not answer questions on self-blame and was excluded from the analyses related to self-blame. The final analytical sample consisted of 200 participants for perceived stigma and 199 participants for self-blame.

### Survey development and outcomes

The questionnaire which was developed as part of the APPROACH study included questions developed by the study investigators in consultation with oncologists and questions taken from validated instruments. The questions were first developed in English (Additional file 1). For this study site, the questionnaire was then translated by professional translators into Vietnamese, and back-translated into English. The original and back-translated English versions were compared, and reconciliations made where necessary. Further revisions were made based on feedback gathered from the physicians and cognitive interviews with ten eligible patients from the study site.

#### Perceived stigma

Perceived stigma was measured by summing the 6 items on sense of stigma from Kissane’s Shame and Stigma Scale and transforming the total score on a scale of 0 to 100 [13]. A total score of 0 indicated no perceived stigma. A higher score indicated a higher level of perceived stigma. This scale displayed good internal consistency with a Cronbach’s alpha of 0.78.

#### Self-blame

Behavioural self-blame was identified by asking “How much do you blame yourself for any behaviour that may have led to your cancer?”. Characterological self-blame was assessed by asking “How much do you blame yourself for the kind of person you are (e.g., being the unlucky person who has things like cancer happen to them)?”. Four options were available: “1-Not at all”, “2-Somewhat”, “3-Very much” and “4-Completely”. The responses were dichotomized into presence (Somewhat/Very much/Completely) or absence (Not at all) of the respective self-blame. In addition, information on patient’s perceived reasons for cancer was collected. The patients were asked to choose from a list that contained the following options: smoking, chewing betel nut/tobacco, consumption of alcohol, being overweight, stress/anxiety, previous bad deeds, God’s will and old age. They were also allowed to specify any other reasons that were not in the list.

#### Depression

Depression was measured via a validated Vietnamese version of the 20-item scale developed by the Center for Epidemiologic Studies (CES-D). Possible score ranged between 0 and 60, with higher CES-D score indicating higher depressive symptoms. A CES-D score of 16 and above suggests the presence of depression [30]. The scale displayed strong reliability with a Cronbach’s alpha of 0.91.

#### Emotional well-being

Emotional well-being (EWB) was measured using the 6-item EWB component of the validated Vietnamese version of the Functional Assessment of Cancer Therapy-General (FACT-G) [31]. Participants were asked to rate their responses on a 5-point scale ranging from 0 to 4. The possible score for EWB ranged between 0 to 24. Higher EWB score indicated better emotional well-being. The EWB component demonstrated good internal consistency with a Cronbach's alpha of 0.84.

#### Social well-being

Social well-being (SWB) was assessed with the 7-item SWB component of FACT-G [31]. This component required participants to rate their responses on a 5-point scale which ranged between 0 and 4. The possible score for SWB ranged between 0 to 28. Higher SWB score indicated better social well-being. This SWB component had a good internal consistency with a Cronbach’s alpha of 0.74.

#### Patient characteristics

Socio-demographic characteristics such as age, sex, marital status, years of education, religion, and cancer type were captured through the survey. Financial distress was assessed by summing three questions [32]: (1) How well does the amount of money you have enable you to cover the cost of your treatment? (2) How well does the amount of money you have enable you to take care of your daily needs? (3) How well does the amount of money you have enable you to buy those little ‘extras’, that is, those small luxuries?. Three possible options were available for these three questions: “0-Very well”, “1-Fairly well”, “2-Poorly”. The possible score for financial distress ranged between 0 to 6, with higher scores reflecting higher financial distress. We also assessed participants’ awareness of disease severity by asking participants to report the current stage of their cancer. Patients who reported advanced stage was considered as having accurate awareness of disease severity. Type of cancer was identified from the medical records.

#### Utilization of and interest in using mental health services

Utilization of mental health services was assessed by asking if patients have seen any of the listed mental health care workers (e.g., psychiatrist, psychologist, medical social worker) as part of their cancer treatment. Interest in using mental health services was determined by asking patients to indicate if they would use mental health services if they were referred.

### Statistical analysis and sample size

Demographic information was summarized with mean and standard deviation (SD) for continuous variables while categorical variables were presented with number, percentage and 95% confidence intervals (95 CI). To investigate the associations, we conducted separate multivariable linear regressions where dependent variables were depressive symptoms (CES-D), emotional well-being (EWB) or social well-being (SWB). The independent variables of interest were perceived stigma, behavioural self-blame or characterological self-blame. These analyses were controlled for patient demographics such as sex (male = 1, female = 0), age, marital status (married = 1, separated/widowed/divorced/never married = 0), years of education, religious affiliation (religious affiliation such as Christian, Buddhist or Taoist = 1, no religious affiliation = 0), financial distress, accurate awareness of disease severity (advanced stage = 1, early stage/don’t know = 0), and cancer type (lung cancer = 1, breast cancer = 1, colorectal cancer = 1, nasopharyngeal cancer = 1, other cancer types = 0).

The sample size of this study was found to be sufficient for the regressions used in this study, assuming an alpha error probability of 0.01 and power of 0.8. To control for Type 1 error due to multiple testing, critical significance level of 0.05 was adjusted according to the Holm’s method [33]. More information on the Holm’s method is available in Additional file 2. All analyses were conducted using Stata 15.

## Results

### Participant characteristics

Demographic information of study participants was tabulated in Table 1. Participants’ ages ranged from 22 to 87 years, with a mean age of 55.2 ± 11.1 years. There were slightly more male (53.5%, *n* = 107) than female participants. The two most common types of cancer among the participants were breast cancer (22.5%, *n* = 45) and lung cancer (22.0%, *n* = 44). Participants received 9.8 ± 3.4 years of education on average. Majority of the participants were married (85.5%, *n* = 171). Slightly less than half (47.0%, *n* = 94) of the participants reported having a religious affiliation. The average reported financial distress was 4.1 ± 2.0 on a scale of 0 to 6. Despite being aware of their cancer diagnosis, about half of the participants (48.5%, *n* = 97) were not aware that they were in the advanced stage of the cancer.

**Table 1:** Demographics of study participants (*N* = 200)

Characteristics	N (%) or mean ± SD
**Age**	55.2 ± 11.1
**Sex**	
Male	107 (53.5%)
Female	93 (46.5%)
**Marital status**	
Married	171 (85.5%)
Separated, widowed, divorced, never married	29 (14.5%)
**Years of education received**	9.8 ± 3.4
**Having a religious affiliation**	94 (47.0%)
**Cancer type**	
Breast cancer	45 (22.5%)
Lung cancer	44 (22.0%)
Nasopharyngeal cancer	21 (10.5%)
Colorectal cancer	20 (10.0%)
Other cancers ^a^	70 (35.0%)
**Financial distress**	4.1 ± 2.0
**Accurate awareness about disease severity (i.e. being at advanced stage)**	
Aware	103 (51.5%)
Not aware	97 (48.5%)
**Perceived reasons for cancer**	
Old age	158 (79.0%)
Smoking	109 (54.5%)
Alcohol consumption	102 (51.0%)
God’s will	82 (41.0%)
Stress	44 (22.0%)
Chewing betel nut/tobacco	33 (16.5%)
Being overweight	21 (10.5%)
Previous bad deeds	13 (6.5%)
Other reasons ^b^	15 (7.5%)
**Perceived stigma**	
Prevalence	158 (79.0%)
Score	20.5 ± 18.0
**Prevalence of behavioural self-blame**	112 (56.3%)
**Prevalence of characterological self-blame**	124 (62.3%)
**Depression (CES-D)**	
Patients with CES-D above 16	133 (66.5%)
Score	20.2 ± 9.5
**Emotional well-being**	13.3 ± 5.6
**Social well-being**	21.6 ± 4.5
**Utilization of mental health services**	
Ever used	6 (3.0%)
Never used	174 (87.0%)
Not sure	20 (10.0%)
**Interest in using mental health services should they be referred**	
Interested	47 (24.2%)
Not interested	56 (28.9%)
Not sure	91 (46.9%)

^a^Other cancers included cancer at bladder, brain, cervical, gastric, kidney, liver, oesophageal, ovarian, oral, pancreas, prostate, soft palate, parotid gland, submandibular gland, melanoma, thymus, bile ducts or ampulla of Vater

^b^Other perceived reasons for cancer included genetic factors, environmental pollution, exposure to chemical substance including insecticide, type of food consumed, and consumption of contaminated food

More than three-fourths (79.0%, *n* = 158) of the participants reported perceived stigma. On a scale of 0 to 100 where higher score reflects higher perceived stigma, the average score for perceived stigma was 20.5 ± 18.0. More than half of the participants reported behavioural self-blame (56.3%, *n* = 112) or characterological self-blame (62.3%, *n* = 124). The top reasons cited as contributing factors of their disease included old age (79.0%, *n* = 158), smoking (54.5%, *n* = 109), alcohol consumption (51.0%, *n* = 1 02) and God’s will (41.0%, *n* = 82).

The average depressive symptoms score was 20.2 ± 9.5 (out of 60), and two-thirds of the participants (66.5%, *n* = 133) reported a score of 16 and above, indicative of potential presence of depression. The average emotional well-being score of the study participants were 13.3 ± 5.6 (out of 24). The average social well-being score was 21.6 ± 4.5 (out of 28).

Only six participants (3.0%) reported having received mental health services while 20 participants (10.0%) were not sure if they have received mental health services before. Among the 194 participants who have never used or are not sure if they have used mental health services, about one-quarter (24.2%, *n* = 47) indicated interest to use mental health services if they were referred.

### Associations of perceived stigma and self-blame with depression, emotional well-being and social well-being

Consistent with our hypothesis, participants who reported higher perceived stigma had lower emotional well-being (ß = -0.1). However, our analyses showed no associations of perceived stigma with depressive symptoms and with social well-being (Table 2).

**Table 2:** Associations of perceived stigma, behavioural self-blame, characterological self-blame with depression, emotional well-being, and social well-being^a^

	**Coefficient** **(ß)**	**95% confidence interval (95 CI)**	***p*-value**
**Perceived stigma**			
Model 1: Depressive symptoms	0.1	0.1, 0.2	0.000
Model 2: Emotional well-being	-0.0 *	-0.1, 0.0	0.024
Model 3: Social well-being	0.0	-0.1, 0.0	0.098
**Presence of behavioral self-blame**			
Model 4: Depressive symptoms	1.7	-1.0, 4.4	0.225
Model 5: Emotional well-being	-1.4	-3.1, 0.2	0.088
Model 6: Social well-being	-0.4	-1.7, 0.9	0.538
**Presence of characterological self-blame**			
Model 7: Depressive symptoms	3.0 *	0.5, 5.5	0.020
Model 8: Emotional well-being	-1.6 *	-3.1, -0.1	0.038
Model 9: Social well-being	0.8	-0.4, 2.0	0.191

^a^Multivariable linear regressions controlled for gender, age, marital status, education, religion, financial distress, awareness of disease severity, and type of cancer

^*^denotes statistical significance after the Holm’s adjustment

Contrary to our hypotheses regarding behavioural self-blame, the findings showed no significant associations of behavioural self-blame with depressive symptoms, emotional well-being and social well-being. On the other hand, participants who experienced characterological self-blame reported greater depressive symptoms (ß = 2.7) and lower emotional well-being (ß = -1.8). However, no significant association was found between social well-being and characterological self-blame. Full list of estimates is available in Additional file 3.

## Discussion

Our study showed that more than half of the Vietnamese advanced cancer patients in our sample reported perceived stigma, behavioural self-blame or characterological self-blame. Although the perceived stigma score reported by our study population may seem low, the figure was higher than those reported by cancer patients in other studies [13, 34]. Our findings also showed that perceived stigma was significantly associated with lower emotional well-being, consistent with other studies [12, 35].

Our findings showed that the relationship between self-blame and psychological outcomes depended on the type of blame, where only characterological self-blame was associated with greater depressive symptoms and lower emotional well-being. This finding is consistent with the Theory of Learned Helplessness which suggests that the perceived lack of control over an outcome such as cancer may affect an individual’s emotions and mental health negatively [36, 37]. The theory suggests that characterological self-blame is more detrimental because it is related to attributes that are relatively non-modifiable or non-controllable [37].

Based on these findings, intervention strategies to reduce self-blame may consider addressing characterological reasons. Although our findings on self-blame are consistent with some studies [19, 38], others reported that both types of self-blame were associated with poorer psychological outcomes [39–41]. The mixed findings in the literature suggest that cultural or other factors might be moderating the relationship between behavioural self-blame and psychological outcomes. This could be a topic for future studies.

Contrary to our hypothesis, social well-being was not significantly associated with perceived stigma, behavioural or characterological self-blame. This could be related to the relatively high social capital and social trust among Vietnamese as compared to countries with similar economic development [42]. In addition, as a society that is deeply rooted in Confucian philosophy, Vietnamese hold firm to the teaching that parents are to be respected regardless of their qualities or faults. Similarly, parents are expected to do their best for their children even at the expense of their own well-being [42]. These cultural norms might have prevented social well-being to decrease for cancer patients even when experiencing cancer stigma or self-blame.

Lastly, the fact that two-thirds of the participants reported a CES-D score of 16 and above, an indicator of possible depression, is worth noting. Equally alarming is the extremely low utilization rate of mental health services among this study population and the relatively low interest in using mental health services. Studies have reported that individuals experiencing a mental condition such as depression are not likely to seek help due to stigmatization and fear for discrimination [43–45]. Due to the lack of resources or lack of help-seeking behaviour, these individuals might not receive the necessary intervention to address their depression [46]. In view of this, it is important to ensure that the psychological needs of advanced cancer patients in Vietnam are adequately addressed through the provision of mental health services. This also echoes the calls of Lancet Commission on Palliative Care and Pain Relief Study Group to alleviate serious health-related suffering among terminally ill patients in low- and middle- income countries like Vietnam and reinforces the urgency of integrating an affordable Essential Package for palliative care into the national health system, which includes interventions to address the psychological needs of terminally ill patients [47].

### Strengths and limitations

To the best of our knowledge, this study is the first to show presence of perceived stigma, behavioural self-blame and characterological self-blame among Vietnamese advanced cancer patients. There are several limitations to this study. First, self-blame was not assessed using an instrument that has been psychometrically validated. Second, we measured behavioural self-blame and characterological self-blame as dichotomous variables (presence versus absence of self-blame). However, the associations of self-blame with depression, and emotional and social well-being may vary based on the intensity of self-blame. Third, since 69 participants in this study reported both behavioural and characterological self-blame, it is possible that we were not able to separate the effects of behavioural self-blame and characterological self-blame on the psychosocial outcomes. Fourth, although the participants in this study were recruited from Hue Central Hospital which is one of the largest hospitals in the country, we acknowledge the possibility that the results may not be generalizable to patients who live in the rural areas of Vietnam. Lastly, as this was a cross-sectional study, our results only imply associations but not causations.

### Implications

Our results demonstrated that patients who reported perceived stigma and characterological self-blame reported greater depressive symptoms and lower emotional well-being, reiterating the importance of assessing and addressing these issues. Although the coefficient for the association between CES-D score and characterological self-blame was not large, any additional increase in CES-D score should be given attention as the mean CES-D score of this population suggests presence of depression. It is also noteworthy that the effect sizes of the associations of characterological social blame with emotional well-being and social well-being were considered moderate based on a meta-analysis on the clinical relevance of changes in these scales [48]. Healthcare professionals involved in the direct care of advanced cancer patients are urged to include mental health assessment to screen for these issues as part of clinical routine. Our results suggest that patients who experience perceived stigma or characterological self-blame can benefit from interventions that address negative inner thoughts and teach coping mechanisms surrounding the issue of cancer stigma and characterological self-blame. Future studies should identify the predictors for individuals who are more susceptible to cancer-related perceived stigma and characterological self-blame amongst cancer patients in Vietnam. Knowing this allows not only health care providers to identify this vulnerable population, but also allows policy makers to design targeted approaches to reduce perceived stigma and self-blame among cancer patients in Vietnam.

## Conclusion

This study serves as an initial situational analysis for policy makers and healthcare providers by providing evidence on the presence of perceived stigma, behavioural self-blame or characterological self-blame among Vietnamese advanced cancer patients. The findings show evidence that perceived stigma was associated with lower emotional well-being, and characterological self-blame was associated with higher depressive symptoms and lower emotional well-being. Early identification of patients who are affected by perceived stigma or self-blame is important to ensure that relevant interventions are offered to these patients in a timely manner.

## Supplementary Information

**Additional file 1.** Survey instrument

**Additional file 2.** Holm’s correction for the *p*-values

**Additional file 3.** Full list of estimates

## Data Availability

The datasets used and/or analysed during the current study are available from the corresponding author on reasonable request.

## Abbreviations

IARC: International Agency for Research on Cancer
APPROACH: Asian Patient Perspectives Regarding Oncology Awareness, Care and Health
NUS-IRB: National University Singapore-Institutional Review Board
FACT-G: Functional Assessment of Cancer Therapy-General
CES-D: Center for Epidemiologic Studies Depression scale
EWB: Emotional well-being
SWB: Social well-being
SD: Standard deviation
CI: 95% Confidence intervals
