# Supplementary material for: The prevalence of perceived stigma and self-blame and their associations with depression, emotional well-being and social well-being among advanced cancer patients: evidence from the APPROACH cross-sectional study in Vietnam
Source: BMC Palliat Care. 2021 Jul 7;20:104. doi: 10.1186/s12904-021-00803-5 (PMC8265020; doi:10.1186/s12904-021-00803-5)
Supplement: Supplementary file 1 — Additional file 1. Survey instrument [file 12904_2021_803_MOESM1_ESM.pdf]

Asian Patient Perspectives Regarding Oncology Awareness, Care, and Health (APPROACH)

For interviewer: Fill in the following information after you have completed the questionnaire.

PARTICIPANT CODE: (FROM CONSENT FORM)

DATE OF INTERVIEW (DD/MM/YYYY): ___/___/_____

TIME STARTED: TIME ENDED: TOTAL INTERVIEW TIME:

INTERVIEWER NAME:

COUNTRY: INTERVIEW LANGUAGE:

NAME OF THE INSTITUTION:

PATIENT TYPE: ☐1 OUTPATIENT ☐2 INPATIENT

SITE OF RECRUITMENT:

☐1 DEPARTMENT OF MEDICAL ONCOLOGY
☐5 DEPARTMENT OF SURGERY
☐6 INTERNATIONAL MEDICAL DEPARTMENT
☐7 DEPARTMENT OF RADIATION ONCOLOGY
☐8 PALLIATIVE CARE UNIT
☐9 OTHER, ___________________________________
☐10 ONCOLOGY DEPARTMENT, HUE CENTRAL HOSPITAL BASE 2

**Fill in the information from patient's medical records.**	PATIENT GENDER: ☐1 MALE ☐2 FEMALE
	PATIENT'S DATE OF BIRTH (DD/MM/YYYY):____/_____/_____
	TYPE OF CANCER: ______________________________

INTRODUCTION

We are conducting a survey to understand the quality of life of patients, quality of care they are currently receiving and their treatment preferences. Your opinions are important to the success of this study. The survey usually takes about **45 minutes**.
There are no right or wrong answers to the questions and you do not have to respond to any questions that you feel uncomfortable answering. Your identity and the information given will be kept strictly confidential and only group data will be reported.

SECTION S: SCREENER

S1 Have you ever been diagnosed with any of the following health conditions? Check all that apply.

	**Health Conditions**	**Yes**
☐1	Diabetes	☐
☐2	Heart conditions (e.g. heart attack, blocked blood vessels)	☐
☐3	Lung/Liver disease (e.g. bronchitis, hepatitis)	☐
☐4	Cancer	☐

[TERMINATE if option 4 'Cancer' is NOT checked]
[Thank you for your interest, but you are not eligible to continue with this survey]

SECTION A: BASIC INFORMATION

[For questions A1 to A5, you do not need to read the response choices out loud to the patient. Allow the patient to first respond directly, and prompt him/her with relevant choices depending upon his/her response]

Refuse to answer

A1 **What is your age?**

☐ **years old** ☐ 888

A2 **How many years of education have you completed (including higher education)?**

☐ **years** ☐ 888

A3 **What is your current marital status?** ☐ 888

☐1	Married	☐4	Divorced
☐2	Separated	☐5	Never married
☐3	Widowed	☐6	Other, specify: _______________

A4 **What is your religion?** ☐ 888

☐1	Hindu	☐7	Jewish
☐2	Muslim	☐8	Parsi/Zoroastrian
☐3	Christian (including Roman Catholic, Protestant, Orthodox, other)	☐9	Taoist
☐4	Sikh	☐10	Confucian
☐5	Buddhist	☐11	No religion
☐6	Jain	☐12	Other, specify: _______________

**A4.3**	**What is your ethnicity?**			
	☐30	Kinh	☐33	Mường
	☐31	Tày	☐17	Others
	☐32	Thái		
**A5**	**What type of cancer have you been diagnosed with?**			
	☐1	Bladder	☐11	Lung
	☐2	Brain	☐12	Nasopharyngeal
	☐3	Breast	☐13	Oesophageal
	☐4	Cervical	☐14	Ovarian
	☐5	Colorectal	☐15	Oral
	☐6	Endometrial	☐16	Pancreatic
	☐7	Gastric	☐17	Prostate
	☐8	Intestinal	☐18	Vulva
	☐9	Kidney	☐19	Others, please specify ________
	☐10	Liver	☐20	Don't know

888

888

**A6**	**Do you know the current stage (i.e. severity) of your cancer?**	
	☐1	Early Stage (Stage I, II or III)
	☐2	Advanced Stage (Stage IV)
	☐3	I don't know
**A7**	**[If A6=2] What was the stage (i.e. severity) of your cancer when you <u>first</u> learned about it?**	
	☐1	Early Stage (Stage I, II or III)
	☐2	Advanced Stage (Stage IV)
	☐3	I don't know

888

888

SECTION B: QUALITY OF LIFE

Refuse to answer

B1[1] **Below is a list of statements that other people with your illness have said are important or bothersome for them. Some of the items may appear to be repetitive, but please bear with us. Please indicate one number per line as it applies to the past 7 days.**

		Not at all	A little bit	Some-what	Quite a bit	Very much	Refuse to answer
B1.1 GP3	Because of my physical condition, I have trouble meeting the needs of my family	0	1	2	3	4	☐ 888
B1.2 GP5	I am bothered by side effects of treatment	0	1	2	3	4	☐ 888
B1.3 GP6	I feel ill	0	1	2	3	4	☐ 888
B1.4 GP7	I am forced to spend time in bed	0	1	2	3	4	☐ 888

		Not at all	A little bit	Some-what	Quite a bit	Very much	Refuse to answer
B1.5 GS1	I feel close to my friends	0	1	2	3	4	☐ 888
B1.6 GS2	I get emotional support from my family	0	1	2	3	4	☐ 888
B1.7 GS3	I get support from my friends	0	1	2	3	4	☐ 888
B1.8 GS4	My family has accepted my illness	0	1	2	3	4	☐ 888
B1.9 GS5	I am satisfied with family communication about my illness	0	1	2	3	4	☐ 888
B1.10 GS6	I feel close to my partner (or the person who is my main support)	0	1	2	3	4	☐ 888
	Regardless of your current level of sexual activity, please answer the following question. If you prefer not to answer it, please mark this box ☐ *and go to the next question.*						
B1.11 GS7	I am satisfied with my sex life	0	1	2	3	4	

[1] Items in B1 are from the Functional Assessment of Chronic Illness Therapy system of Quality of Life questionnaires ("FACIT system"). Items B1.1–B1.24 are from FACT-G (Version 4), Items B1.25–B1.36 are from FACIT-Sp (Version 4) and B1.37 from FACIT-Pal (Version 4).

		Not at all	A little bit	Some-what	Quite a bit	Very much	
B1.12 GE1	I feel sad	0	1	2	3	4	888

		Not at all	A little bit	Some-what	Quite a bit	Very much	Refuse to answer
B1.13 GE2	I am satisfied with how I am coping with my illness	0	1	2	3	4	888
B1.14 GE3	I am losing hope in the fight against my illness	0	1	2	3	4	888
B1.15 GE4	I feel nervous	0	1	2	3	4	888
B1.16 GE5	I worry about dying	0	1	2	3	4	888
B1.17 GE6	I worry that my condition will get worse	0	1	2	3	4	888

		Not at all	A little bit	Some-what	Quite a bit	Very much	
B1.18 GF1	I am able to work (include work at home)	0	1	2	3	4	888
B1.19 GF2	My work (include work at home) is fulfilling	0	1	2	3	4	888
B1.20 GF3	I am able to enjoy life	0	1	2	3	4	888
B1.21 GF4	I have accepted my illness	0	1	2	3	4	888
B1.22 GF5	I am sleeping well	0	1	2	3	4	888
B1.23 GF6	I am enjoying the things I usually do for fun	0	1	2	3	4	888
B1.24 GF7	I am content with the quality of my life right now	0	1	2	3	4	888

		Not at all	A little bit	Some-what	Quite a bit	Very much	
B1.25 Sp1	I feel peaceful	0	1	2	3	4	888
B1.26 Sp2	I have a reason for living	0	1	2	3	4	888
B1.27 Sp3	My life has been productive	0	1	2	3	4	888

B1.28 Sp4	I have trouble feeling peace of mind	0	1	2	3	4	☐ 888 Refuse to answer
B1.29 Sp5	I feel a sense of purpose in my life	0	1	2	3	4	888
		**Not at all**	**A little bit**	**Some-what**	**Quite a bit**	**Very much**	
B1.30 Sp6	I am able to reach down deep into myself for comfort	0	1	2	3	4	☐ 888
B1.31 Sp7	I feel a sense of harmony within myself	0	1	2	3	4	☐ 888
B1.32 Sp8	My life lacks meaning and purpose	0	1	2	3	4	☐ 888
B1.33 Sp9	I find comfort in my faith or spiritual beliefs	0	1	2	3	4	☐ 888
B1.34 Sp10	I find strength in my faith or spiritual beliefs	0	1	2	3	4	☐ 888
B1.35 Sp11NI	Difficult times have strengthened my faith or spiritual beliefs	0	1	2	3	4	☐ 888
B1.36 Sp12NI	Even during difficult times, I know that things will be okay	0	1	2	3	4	☐ 888
B1.37 Sp21	I feel hopeful	0	1	2	3	4	☐ 888

The next three questions will focus on your finances.

**B2**	**How well does the amount of money you have enable you to cover the cost of your treatment?**		☐ 888
	☐1	Very well	
	☐2	Fairly well	
	☐3	Poorly	
**B3**	**How well does the amount of money you have take care of your daily needs?**		☐ 888
	☐1	Very well	
	☐2	Fairly well	
	☐3	Poorly	
**B4**	**How well does the amount of money you have enable you to buy those little 'extras', that is, those small luxuries?**		☐ 888
	☐1	Very well	
	☐2	Fairly well	

	☐3	Poorly

**B10.1**	**In the last 24 hours, have you taken any medication for pain relief?**	
	☐1	Yes
	☐2	No

888

Refuse to answer

B13 [2] **Below is a list of symptoms that people with your illness commonly experience. Please circle or mark one number per line to indicate your response as it applies to the past 7 days.**

	**SYMPTOM MANAGEMENT**	**Not at all**	**A little bit**	**Some-what**	**Quite a bit**	**Very much**	
B13.1 GP4	I have pain	0	1	2	3	4	888
B13.2 B1	I have been short of breath	0	1	2	3	4	888
B13.3 PAL5	I am constipated	0	1	2	3	4	888
B13.4 C2	I am losing weight	0	1	2	3	4	888
B13.5 O2	I have been vomiting	0	1	2	3	4	888
B13.6 PAL6	I have swelling in parts of my body	0	1	2	3	4	888
B13.7 PAL7	My mouth and throat are dry	0	1	2	3	4	888
B13.8 GP1	I have lack of energy	0	1	2	3	4	888
B13.9 GP2	I have nausea	0	1	2	3	4	888
B13.10	Any other symptom, please specify: ________________		1	2	3	4	888

**B14**	**(If any of B13.1 to B13.10 ≠0) Did you tell your doctor about your symptoms?**	
	☐1	Yes
	☐2	No

888

[2] Items B13.1–B13.6 are from FACIT-Pal (Version 4) and B13.8–B13.9 are from FACT-G (Version 4). Both FACIT-Pal and FACT-G are from the Functional Assessment of Chronic Illness Therapy system of Quality of Life questionnaires ("FACIT system").

B15	**(If B14=2) Why did you not tell your doctor about your symptoms? Check all that apply.**
☐1	I can manage my symptoms myself
☐2	I want the doctor to focus on the treatment of my cancer
☐3	I did not get an opportunity to tell the doctor
☐4	I believe that it is easier to put up with symptoms than with the side effects that come from medicines to treat these symptoms.
☐5	I will use medicines only as a last resort to treat my symptoms
☐6	I believe that good patients avoid talking about symptom/s
☐7	I can get addicted easily to medicines needed to treat these symptoms
☐8	I believe that tolerating symptoms builds character—it's good for me.
☐9	Any other reason, specify____
☐10	I believe that tolerating pain and symptoms would help me get rid of my sins.

888

B16.15[3] **Below is a list of the ways you might have felt or behaved. For each of the following statements, please choose the option that best describes how often you felt or behaved this way during the PAST WEEK.**

		Rarely or none of the time (Less than 1 day)	Some or a little of the time (1-2 days)	Occasionally or a moderate amount of the time (3-4 days)	Most or all of the time (5-7 days)
B16.15.1	I was bothered by things that usually don't bother me.	1	2	3	4
B16.15.2	I did not feel like eating; my appetite was poor.	1	2	3	4

888

888

[3] Items from B16.15 are from the CES-D 20-item scale. Radloff, L.S. (1977). The CES-D scale: A self-report depression scale for research in the general population. Applied psychological measurement, 1(3), 385-401.

B16.15.3	I felt that I could not shake off the blues even with help from my family or friends.	1	2	3	4	888
B16.15.4	I felt that I was just as good as other people.	1	2	3	4	Refuse to answer 888
B16.15.5	I had trouble keeping my mind on what I was doing.	1	2	3	4	888 888
B16.15.6	I felt depressed.	1	2	3	4	888 888
B16.15.7	I felt that everything I did was an effort.	1	2	3	4	888
B16.15.8	I felt hopeful about the future.	1	2	3	4	888
B16.15.9	I thought my life had been a failure.	1	2	3	4	888
B16.15.10	I felt fearful.	1	2	3	4	888
B16.15.11	My sleep was restless.	1	2	3	4	888
B16.15.12	I was happy.	1	2	3	4	888
B16.15.13	I talked less than usual.	1	2	3	4	888
B16.15.14	I felt lonely.	1	2	3	4	888
B16.15.15	People were very unfriendly.	1	2	3	4	888
B16.15.16	I enjoyed life.	1	2	3	4	888
B16.15.17	I had crying spells.	1	2	3	4	888
B16.15.18	I felt sad.	1	2	3	4	888
B16.15.19	I felt that people dislike me.	1	2	3	4	888
B16.15.20	I could not get “going”.	1	2	3	4	888

B17 I feel like I have become a burden on my loved ones. 888

☐1	Not at all
☐2	A little bit
☐3	Somewhat

	☐4	Quite a bit
	☐5	Very much

SECTION C: QUALITY OF CARE

Refuse to answer

C1[4] **Now we would like to ask you some questions related to your experience in dealing with health care providers for your current illness. Think of all the healthcare providers (doctors, nurses, and allied health providers) that you have seen for your current illness since it was diagnosed. These include those in the hospital, polyclinics, private clinics, hospice, nursing homes or other places.**

		Always (1)	Sometimes (2)	Never (3)	Not applicable (4)	Refuse to answer
C1.1	How often did your doctors listen carefully to you?	☐	☐	☐		☐ 888
C1.2	How often did your doctors explain things in a way you could understand?	☐	☐	☐		☐ 888
C1.3	How often did your doctors give you as much information as you wanted about your treatments, including potential benefits and side effects?	☐	☐	☐		☐ 888
C1.4	How often did your doctors encourage you to ask all the cancer related questions you had?	☐	☐	☐		☐ 888
C1.5	How often did your doctor treat you with courtesy and respect?	☐	☐	☐		☐ 888
C1.5.1	How often did your doctors spend adequate time with you to address all your concerns?	☐	☐	☐		
C1.6	How often were your nurses as helpful as you thought they should be?	☐	☐	☐		☐ 888
C1.7	How often did your nurses treat you with courtesy and respect?	☐	☐	☐		☐ 888
C1.8	How often did you think that your health problems related to your cancer or its treatment were handled quickly enough?	☐	☐	☐		☐ 888
C1.9	How often were you able to see the specialist doctors you wanted to see for your cancer?	☐	☐	☐	☐	☐ 888
C1.10	How often did the doctors, nurses, and other medical staff providing your care seem to work well together as a team?	☐	☐	☐		☐ 888

Refuse to answer

**C1.11**	How often did your doctors seem to be aware of treatments for your cancer that other doctors recommended?	☐	☐	☐	☐	☐ 888
**C1.12**	How often did you know who to ask when you had any questions related to your cancer or its treatments?	☐	☐	☐	☐	☐ 888
**C1.13**	How often did you feel that your doctors, nurses, and other medical staff did everything they could to treat your health problems related to your cancer or its treatments?	☐	☐	☐		☐ 888
**C1.14**	How often did you feel you were as much involved in decisions about your care as you wanted?	☐	☐	☐		☐ 888

C1.15 **Overall how would you rate the quality of your health care since your diagnosis?** ☐ 888

☐1	Excellent	☐4	Fair
☐2	Very good	☐5	Poor
☐3	Good		

C2 **Now I would like you to think about your most recent visit again. I want to know your impressions of your most recent visit for health care. I would like you to rate your experiences using the following questions.**

For your last visit to a health care provider, how would you rate the following:

		Very Good	Good	Moderate	Bad	Very Bad	
C2.1	...your experience of being treated respectfully?	1	2	3	4	5	☐ 888
C2.2	…how clearly health care providers explained things to you?	1	2	3	4	5	☐ 888

---

[4] Items from C1 except for C1.5.1 and C1.14 are from CanCORS Full Patient Baseline Interview (Version 7); Ayanian JZ, Zaslavsky AM, Arora NK, Kahn KL, Malin JL, Ganz PA, van Ryn M, Hornbrook MC, Kiefe CI, He Y, Urmie J, Weeks JC, Harrington DP. Patients' experiences with care for lung cancer and colorectal cancer: findings from the Cancer Care Outcomes Research and Surveillance Consortium. J Clin Oncol 2010; 28:4154-61.
Malin JL, Ko C, Ayanian JZ, Harrington D, Kahn KL, Ganther-Urmie J, Catalano PJ, Nerenz DR, Zaslavsky AM, Wallace RB, Guadagnoli E, Arora NK, Roudier MD, Ganz PA. Understanding cancer patients' experiences and outcomes: development and pilot study of the Cancer Care Outcomes Research and Surveillance patient survey. Supp Care Cancer 2006; 14:837-48.

C2.3	…your experience of being involved in making decisions for your treatment?	1	2	3	4	5

888

Refuse to answer

C3[5]	I am now going to read you stories describing experiences that other people with your illness have had with health care services. I want you to think about these people's experiences as if they were your own. Once I have finished reading each story, I will ask you to rate what happened in the story as very good, good, moderate, bad or very bad.					
C3.1	[Tý] went to a crowded clinic. No-one greeted her. She waited for 30 minutes when a nurse called for her for an examination behind a screen that separated the waiting area from the examination area.					
	How would you rate [Tý's] experience of being greeted and talked to respectfully?...	Very Good	Good	Moderate	Bad	Very Bad
		1	2	3	4	5
C3.2	The doctor has very briefly explained to [Tẩn] about his illness. He is very busy and there is a queue of patients waiting to see him. [Tẩn] would like to know more about his illness, but feels that there is no time to ask questions. The doctor says goodbye to [Tẩn], and [Tẩn] leaves the office.					
	How would you rate [Tẩn] experience of how clearly health care providers explained things to him?	Very Good	Good	Moderate	Bad	Very Bad
		1	2	3	4	5
C3.3	The doctor ordered some blood tests and scan for [Hàn]. [Hàn] didn't know why he needed blood tests and scan and was worried until the doctor explained what they were for.					
	How would you rate [Hàn's] experience of being involved in making decisions about his health care or treatment?	Very Good	Good	Moderate	Bad	Very Bad
		1	2	3	4	5

888

888

888

[5] Adapted from WHO SAGE Wave 1 Individual questionnaire obtained from http://www.who.int/healthinfo/sage/cohorts/en/index2.html.

Refuse to answer

SECTION D: PROGNOSIS, TREATMENT PREFERENCES, AND DECISION MAKING

D1 (If A6 ≠ 2) When did you first learn that you have cancer?

				Refuse to answer
☐1	Less than 6 months ago	☐4	2 to 3 years ago	☐ 888
☐2	6 months to 1 year ago	☐5	More than 3 years ago	
☐3	1 to 2 years ago			

D2 (If A6 ≠ 2) Who informed you about it?

				Refuse to answer
☐1	Doctor	☐4	Guessed from the worsened condition	☐ 888
☐2	Family member	☐5	Other, please explain:_______________	
☐3	Learned by chance			

D3 (If A6 = 2) When did you first learn that you have advanced cancer?

				Refuse to answer
☐1	Less than 6 months ago	☐4	2 to 3 years ago	☐ 888
☐2	6 months to 1 year ago	☐5	More than 3 years ago	
☐3	1 to 2 years ago			

D4 (If A6 = 2) Who informed you about it?

				Refuse to answer
☐1	Doctor	☐4	Guessed from the worsened condition	☐ 888
☐2	Family member	☐5	Other, please explain:_______________	
☐3	Learned by chance			

D5[6] How informed are you regarding how your illness will change over time?

				Refuse to answer
☐1	Very informed	☐3	Unsure	☐ 888
☐2	Somewhat informed	☐4	Not informed	

[6] CanCORS Full Patient Baseline Interview (Version 7); Ayanian JZ, Zaslavsky AM, Arora NK, Kahn KL, Malin JL, Ganz PA, van Ryn M, Hornbrook MC, Kiefe CI, He Y, Urmie J, Weeks JC, Harrington DP. Patients' experiences with care for lung cancer and colorectal cancer: findings from the Cancer Care Outcomes Research and Surveillance Consortium. J Clin Oncol 2010; 28:4154-61.

Malin JL, Ko C, Ayanian JZ, Harrington D, Kahn KL, Ganther-Urmie J, Catalano PJ, Nerenz DR, Zaslavsky AM, Wallace RB, Guadagnoli E, Arora NK, Roudier MD, Ganz PA. Understanding cancer patients' experiences and outcomes: development and pilot study of the Cancer Care Outcomes Research and Surveillance patient survey. Supp Care Cancer 2006; 14:837-48.

The next items ask what you think about the possible results of cancer treatments. Do you think …

**D6[6]**	**The current treatments you are taking for your cancer will help you live longer?**	
	☐1	Yes
	☐2	No
	☐3	Not sure
**D7[6]**	**The current treatments you are taking for your cancer will cure you?**	
	☐1	Yes
	☐2	No
	☐3	Not sure
**D8**	**There are other treatments (besides your current treatment) that will help you live longer?**	
	☐1	Yes
	☐2	No
	☐3	Not sure
**D9**	**There are other treatments (besides your current treatment) that will cure you?**	
	☐1	Yes
	☐2	No
	☐3	Not sure
**D10[6]**	**Since diagnosis, who has been responsible for the most important decisions about your treatment? Check all that apply.**	
	☐1	Myself
	☐2	My family
	☐3	My doctors
**D11[6]**	**(If more than one option is checked in D10.) Which statement best describes the role each person played when making decisions about your treatment?**	
	(If "myself" and "my family" are checked, options are)	
	☐1	I made the decisions after considering my family's opinion
	☐2	My family made the decisions after considering my opinion
	☐3	My family and I made the decisions together
	(If "myself" and "my doctors" are checked, options are)	
	☐4	I made the decisions after considering my doctors' opinions
	☐5	My doctors made the decisions after considering my opinion
	☐6	My doctors and I made the decisions together

☐ 888

☐ 888

☐ 888

☐ 888

☐ 888

☐ 888

Refuse to answer

	(If "my family" and "my doctors" are checked, options are)	
	☐7	My family made the decisions after considering my doctors' opinions
	☐8	My doctors made the decisions after considering my family's opinion
	☐9	My family and my doctors made the decisions together
	(If all three are checked, options are)	
	☐10	I made the decisions after considering my family's and doctors' opinions
	☐11	My family made the decisions after considering my and my doctors' opinions
	☐12	My doctors made the decisions after considering my and my family's opinions
	☐13	My family, my doctors and I made the decisions together
**D12** 6	**Since diagnosis, who do you wish had been responsible for the most important decisions about your treatment? Check all that apply.**	
	☐1	Myself
	☐2	My family
	☐3	My doctors
**D13** 6	**(If more than one option is checked in D12.) Which statement best describes the role you wish each person had played when making decisions about your treatment?**	
	(If "myself" and "my family" are checked, options are)	
	☐1	I wish that I made the decisions after considering my family's opinion
	☐2	I wish that my family made the decisions after considering my opinion
	☐3	I wish that my family and I made the decisions together
	(If "myself" and "my doctors" are checked, options are)	
	☐4	I wish that I made the decisions after considering my doctors' opinions
	☐5	I wish that my doctors made the decisions after considering my opinion
	☐6	I wish that my doctors and I made the decisions together
	(If "my family" and "my doctors" are checked, options are)	
	☐7	I wish that my family made the decisions after considering my doctors' opinions
	☐8	I wish that my doctors made the decisions after considering my family's opinion
	☐9	I wish that my family and my doctors made the decisions together
	(If all three are checked, options are)	
	☐10	I wish that I made the decisions after considering my family's and doctors' opinions
	☐11	I wish that my family made the decisions after considering my and my doctors' opinions

888

888

	☐12	I wish that my doctors made the decisions after considering my and my family's opinions
	☐13	I wish that I, my family and my doctors made the decisions together
**D14**	**If there is a difference in opinion between you and your family regarding the appropriate course of treatment for you, whose opinion do you think will be most important in deciding the treatment that you receive?**	
	☐1	My own
	☐2	My family's
	☐3	My doctor's

Refuse to answer

☐ 888

We will now ask you questions about your treatment preferences. ***The next questions that I am going to ask you are very sensitive in nature. Please skip the questions that you do not feel comfortable answering.***

D15[6] **If you had to make a choice now, would you prefer treatment that extends life as much as possible, or would you want treatment that gives you minimal pain and discomfort? Please choose a point in the scale below.**

☐ 888

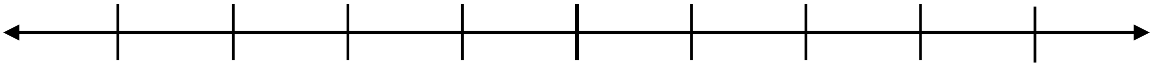

Extend life as much as possible<br>Severe pain or discomfort	Moderate life extension<br>Moderate pain or discomfort	No life extension<br>Minimal pain or discomfort

D16[6] **If you had to make a choice now, would you prefer treatment that extends life as much as possible, or would you want treatment that costs you less? Please choose a point in the scale below.**

☐ 888

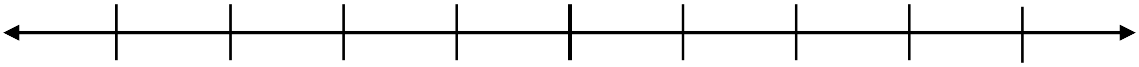

Extend life as much as possible<br>High cost	Moderate life extension<br>Moderate cost	No life extension<br>Less cost

D17 **Have you discussed your treatment and care preferences with your family member(s)?**

☐ 888

☐1	Yes	☐2	No

SECTION E: HOSPICE PALLIATIVE CARE AWARENESS AND UTILIZATION

Refuse to answer

E1[6] **Please indicate if you are aware of hospice palliative care services?** (*Hospice palliative care is a comprehensive program that focuses on providing relief of pain and other distressing symptoms, as well as ensuring emotional, spiritual and practical support for the patient and the family. These services can be given in the patient's home, in an in-patient hospice, hospice day care centre, in a palliative care clinic or in the ward of a hospital)*

☐1	Yes
☐2	No (Go to E8)
☐3	Not sure (Go to E8)

☐ 888

E2 **How did you first learn about hospice palliative care services?**

☐1	From doctors or other healthcare professionals	☐4	From the media
☐2	From family/friends	☐5	From others, please specify:_______________
☐3	Through personal research		

☐ 888

E3[6] **Did any doctor or other health care provider <u>recommend</u> hospice palliative care to you?**

☐1	Yes
☐2	No (Go to E5)
☐3	Not sure (Go to E5)

☐ 888

E4 **When did the doctor recommend hospice palliative care for you?**

☐1	Within the last month	☐4	1 year ago
☐2	2 to 3 months ago	☐5	More than 1 year ago
☐3	4 to 6 months ago		

☐ 888

E5[6] **Since the diagnosis of your illness, have you received hospice palliative care?**

☐1	Yes
☐2	No (Go to E7)
☐3	Not sure (Go to E7)

☐ 888

E6[6] **(If E5=1) Please tell us who had the FINAL say in deciding for you to receive hospice palliative care.**

☐1	Me only	☐5	Me and my doctor
☐2	My family only	☐6	My family and my doctor
☐3	My doctor only	☐7	Me, my family and my doctor
☐4	Me and my family	☐8	Others, please specify:_________

☐ 888

Refuse to answer

**E7**	**[You do not need to read response choices out loud. Allow the patient to first respond directly, and prompt him/her with relevant choices depending upon his/her response]** **(If E5≠1) Why are you not receiving hospice palliative care at this time? Check all that apply.**	☐ 888
	☐1 I am still receiving treatment for my disease	
	☐2 I do not believe it's time for hospice palliative care	
	☐3 I do not think that hospice palliative care would be of help to me	
	☐4 Some of my doctors do not think that hospice palliative care would be of help to me	
	☐5 My family does not believe it's time for hospice palliative care	
	☐6 My family does not think that hospice palliative care would be of help to me	
	☐7 I don't know much about it	
	☐8 I think getting hospice palliative care will be like giving up on life	
	☐9 It is expensive	
	☐10 Any other reason, please specify:________________	
	The next questions that I am going to ask you are very sensitive in nature. We are asking you these questions to better understand your thoughts on your health, quality of life and the quality of care you receive from this hospital. Please skip the questions that you do not feel comfortable answering.	
**E8**	**Would you like to know how long you are likely to live under various treatment options?**	☐ 888
	☐1 No	
	☐2 Yes, in general terms (such as 'a few months' or 'a few years')	
	☐3 Yes, in specific terms (such as 'on average 6 months')	
	☐4 Not Sure	
**E9**	**[You do not need to read response choices out loud. Allow the patient to first respond directly, and prompt him/her with relevant choices depending upon his/her response]** **Considering your current health condition and treatment plan, how long do you think you are likely to live?**	☐ 888
	☐1 Less than 1 year	
	☐2 1 up to 2 years	
	☐3 2 up to 3 years	
	☐4 3 up to 5 years	
	☐5 5 up to 7 years	
	☐6 7 up to 10 years	
	☐7 More than 10 years, please specify _______________________________	

Refuse to answer

**E10**	**Some people have a strong preference concerning where they would like to spend their last days prior to death. If you had a chance, where would you like to be during the last days of your life?**	
	☐1	At my home
	☐2	In the home of a friend/family member
	☐3	In the hospital where I have been receiving medical care
	☐4	Hospice
	☐5	Nursing home
	☐6	Any other place, please specify:_______________
	☐7	Doesn't matter
**E11**[7]	**Do you ever wish that your life would end sooner?**	
	☐1	Yes
	☐2	No
	☐3	Not sure

☐ 888

☐ 888

[7] From the Desire for Death Rating Scale. Chochinov HM, Wilson KG, Enns M, Mowchun N, Lander S, Levitt M, Clinch JJ: Desire for death in the terminally ill. Am J Psychiatry 1995; 152:1185–1191.

Refuse to answer

SECTION F: SELF BLAME AND SOCIAL STIGMA

F1[6] **Have you smoked at least 100 times in your life?**

☐ 1	Yes	**Answer Qs. F1.1 & F1.2**
☐ 2	No	**Skip to Qs. F2**
☐ 3	Don't know	

☐ 888

F1.1[6] **How old were you when you started to smoke regularly?**

☐ years old

☐ 888

F1.2 **Are you currently smoking?**

☐ 1	**Yes**
☐ 2	**No**

☐ 888

F2 **Have you ever chewed betel nut/tobacco regularly?**

☐ 1	Yes, I currently chew betel nut/tobacco regularly
☐ 2	Yes, I have chewed betel nut/tobacco in the past but not anymore
☐ 3	No

☐ 888

F2.1 **(If F2= 1 or 2) How old were you when you started to chew betel nut/tobacco regularly?**

☐ years old

☐ 888

F3[6] **Before you were diagnosed with cancer, how often did you drink any type of alcoholic beverage?**

☐ 1	Less than once a month
☐ 2	A few times a month
☐ 3	A few times a week
☐ 4	Daily

☐ 888

F3.1[6] **(If F3≠1) Before you were diagnosed with cancer, how much alcohol did you usually have each time you had drinks?**

☐ 1	1 – 2 drinks
☐ 2	3 – 4 drinks
☐ 3	5 or more drinks

☐ 888

F3.2[6] **Are you currently consuming any alcoholic beverages?**

☐1	Yes	☐2	No

☐ 888

Refuse to answer

F4 ***We have found that some people blame themselves for their cancer and some people don't blame themselves at all.***
How much do you blame yourself for:

		Not at all (1)	Somewhat (2)	Very much (3)	Completely (4)	
i.	Any behaviour that may have led to your cancer?	☐	☐	☐	☐	888
ii.	The kind of person you are (e.g., being the unlucky person who has things like cancer happen to them)?	☐	☐	☐	☐	888

F5 **To the extent of your knowledge, which of the following (if any) are reasons for the type of illness you have? Check all that apply.** 888

1	Smoking	☐
2	Chewing betel nut/tobacco	☐
3	Consumption of alcohol	☐
4	Being overweight	☐
5	Stress/ Anxiety	☐
6	Previous bad deeds	☐
7	God's will	☐
8	Old age	☐
9	Others, please specify ________________________	

F6[8] **We have found that some people feel differently about themselves and experience changes in their social interactions due to their cancer. Consider the statements below and indicate how often they happen for you.**

		Never (1)	Rarely (2)	Sometimes (3)	Often (4)	Always (5)	
i.	I feel others consider me responsible for my cancer.	☐	☐	☐	☐	☐	888
ii.	I am embarrassed when I tell people my diagnosis	☐	☐	☐	☐	☐	888
iii.	I feel ashamed for having developed cancer	☐	☐	☐	☐	☐	888
iv.	People avoid me because of my cancer	☐	☐	☐	☐	☐	888
v.	I have an urge to keep my cancer a secret	☐	☐	☐	☐	☐	888
vi.	I sense that others feel strained when they are around me.	☐	☐	☐	☐	☐	888

[8] Item is from the Shame Stigma Scale. Kissane DW, Patel SG, Baser RE, Bell R, Farberov M, Ostroff JS, et al. Preliminary evaluation of the reliability and validity of the shame and stigma scale in head and neck cancer. Head Neck. 2013;35:172-83.

Refuse to answer

**F7**	**Do you think your family and friends think cancer is contagious?**			
	☐ 1	Yes, most of them	☐ 3	No
	☐ 2	Yes, some of them	☐ 4	Not sure

888

**F8**	**How much control do you believe you personally have over:**				
		**Absolutely no control (1)**	**Little bit of control (2)**	**Some control (3)**	**Total Control (4)**
**i.**	The stress from your illness?	☐	☐	☐	☐
**ii.**	Physical symptoms of your disease or side effects of your treatment (e.g., pain)?	☐	☐	☐	☐
**iii.**	The type of medical treatment you receive?	☐	☐	☐	☐
**iv.**	Whether your condition will get better?	☐	☐	☐	☐

888

888

888

888

**F9**	**Do you believe God will cure your illness one day?**	
	☐ 1	Yes
	☐ 2	No
	☐ 3	Not sure

888

**F9.1**	**[If F9=Yes] How will this happen?**	
	☐ 1	Through a miracle without medical treatment
	☐ 2	Through medical treatment

888

SECTION G: USE OF/INTEREST IN USING MENTAL HEALTH SERVICES

Refuse to answer

**G1**	**Have you seen any of the mental health care workers listed below as part of your cancer treatment? Check all that apply.**				☐ 888
	☐ 1	Psychiatrist	**Answer Qs. G2 to G4**		
	☐ 2	Psychologist			
	☐ 3	Medical social worker (for psychological support)			
	☐ 4	Community counsellor			
	☐ 5	Others, please specify ________________			
	☐ 6	Don't know	**Skip to Q. G5**		
	☐ 7	No			
**G2**	**[If G1= 1 to 5] What type of mental health service did you receive?**				☐ 888
	☐ 1	Medications	☐ 3	Support group	
	☐ 2	Therapy/counselling	☐ 4	Other, please specify:_______________	
**G3**	**[If G1= 1 to 5] Where did you receive mental health services?**				☐ 888
	☐1	At the hospital as part of inpatient treatment			
	☐2	At the hospital in an outpatient appointment			
	☐3	I found a mental health professional myself			
	☐4	As part of homecare service			
**G4**	**[If G1= 1 to 5] How helpful did you find receiving mental health services?**				☐ 888
	☐ 1	Very helpful	☐ 3	I am not sure	
	☐ 2	Quite helpful	☐ 4	Not helpful at all	
**G5**	**[If G1= 6 or 7] Would you use mental health services if you were referred?**				☐ 888
	☐ 1	Yes			
	☐ 2	No			
	☐ 3	Not sure			
**G6**	**Did you delay medical treatment for cancer for any reason?**				☐ 888
	☐ 1	Yes, I delayed my medical treatment for some time	**Answer Q. G6.1**		
	☐ 2	No, I sought medical treatment immediately	**Skip to Section H**		

Refuse to answer

G6.1	[If G6=1] What were the reasons for delay in your treatment? Check all that apply.		
	☐1	Trouble in identifying a suitable treatment plan	☐ 888
	☐2	Cost of treatment	
	☐3	Using alternative methods of healing before medical treatment (e.g., herbal treatment, prayer, homeopathy, diet therapy)	
	☐4	I felt overwhelmed/confused and did not know what to do	
	☐5	I felt ashamed to get help for my illness	
	☐6	My family did not think that I need medical treatment	
	☐7	Other reasons, please specify ________________________________	

SECTION H: USE OF COMPLEMENTARY AND ALTERNATIVE THERAPY

Complementary and Alternative Therapies refer to treatments that are used either (1) together with medical treatment, or (2) instead of medical treatment. Examples include herbal treatment, diet therapy, exercise (e.g., qi gong, yoga) and spiritual healing.

H1[6]	Since diagnosis of cancer, what type of complementary and alternative therapy have you used? Check all that apply.			
	☐1	Traditional Chinese Medicine		☐ 888
	☐2	Traditional Indian Medicine (e.g., Ayurveda, unani, siddha)		
	☐3	Homeopathy		
	☐4	Western herbal/health supplements		
	☐5	Others, please specify _________________________________		
	☐6	None of above		
	☐8	Traditional Vietnamese Medicine		
**H2[6]**	**Have you used any of the therapies listed below for your cancer? Check all that apply.**			
	☐1	Herbal medicine to consume	**Answer Qs. H2.1 to H2.6**	☐ 888
	☐2	Diet therapy (e.g., macrobiotic diet)		
	☐3	Acupuncture/ acupressure/ moxibustion		
	☐4	Massage/ Reflexology		
	☐5	Exercise (e.g., qi gong, tai chi, yoga)		
	☐6	Spiritual/faith healing by others		
	☐7	Others, please describe ___________________		
	☐8	None of above	**Skip to next section**	
**H2.1**	**[If H2=1 to 7] When did you start using this therapy for cancer?**			Refuse to answer
	☐1	Before being treated in the hospital		☐ 888

	☐2	While I was being treated in the hospital			
	☐3	After being treated in the hospital			
**H2.2**	**[If H2=1 to 7] Is the therapy meant to replace your medical treatment?**				888
	☐ 1	Yes	☐ 2	No	
**H2.3[6]**	**[If H2=1 to 7] Did you tell your doctor that you are using this therapy for your cancer?**				888
	☐ 1	Yes			
	☐ 2	No			
**H2.3.1**	**[If H2.3=1] How supportive was your doctor of this therapy?**				888
	☐ 1	Supportive	☐ 3	Neutral	
	☐ 2	Not supportive	☐ 4	Don't know	
**H2.4[6]**	**[If H2=1 to 7] What is the main reason for using this therapy?**				888
	☐1	To boost my immune system			
	☐2	To reduce side effects of treatment			
	☐3	To control my symptoms (e.g. pain). If so, describe the symptom ________________			
	☐4	To prolong life			
	☐5	To cure my illness			
	☐6	Others. Please describe ____			
**H2.5**	**[If H2=1 to 7] How long have you been using this therapy?**				888
	☐1	Less than 1 month			
	☐2	Between 1 to 3 months			
	☐3	Between 3 to 6 months			
	☐4	More than 6 months			
	☐5	I am no longer using this therapy			
**H2.6[6]**	**[If H2=1 to 7] Roughly how much did you spend, in total, on complementary and alternative therapies in the last 3 months?** ___________________________				888

SECTION I: DEMOGRAPHICS

Refuse to answer

**I1**	**What is your current occupation?**			
	☐1	Farmer	☐6	Service- Government
	☐2	Wage labourer	☐7	Homemaker
	☐3	Skilled worker	☐8	Retired
	☐4	Shop keeper	☐9	Unemployed
	☐5	Self-employed	☐10	Others, please specify _________
**I2**	**Were you working before you were seeking care at this hospital, for your illness?**			
	☐1	Working full-time	☐4	Homemaker
	☐2	Working part-time	☐5	Unemployed
	☐3	Retired and not working		
**I3**	**How many persons live in your household?**			
	☐	Number of people		
**I4**	**How would you rate the economic status of your household?**			
	☐1	Poor	☐3	Upper Middle Class
	☐2	Lower Middle Class	☐4	Wealthy

888

888

888

888

Thank you for completing the survey.

POST INTERVIEW: OBSERVATIONS OF THE INTERVIEWER

[Interviewer: Please fill in the following questions based on your observations during the interview.]

INT1 During the survey, was there someone either present in the room or in a room nearby who could hear the contents of the interview?

☐1	Yes, during most of the interview
☐2	Yes, during half of the interview
☐3	Yes, at times during the interview
☐4	For the most part, no other person was present to hear

INT2 To what extent did this person influence the patient's responses?

☐1	Would correct the patient's responses or prevent the patient from giving his or her own responses
☐2	Listened to the interview, but did not interrupt verbally
☐3	Hardly paid any attention to the interview
☐4	Didn't seem to have any effect on the patient's responses

INT3 The following concerns your impression of the patient.

		Yes (1)	Some what (2)	Not really (3)	No (4)	Not sure (5)
**i.**	Did you feel that the patient was mentally competent enough to provide adequate responses?	☐	☐	☐	☐	☐
**ii.**	Did you feel that the patient understood the questions?	☐	☐	☐	☐	☐
**iii.**	Did you feel that the patient was responsive to the interview?	☐	☐	☐	☐	☐
**iv.**	Did you feel the patient's hearing/visual difficulties adversely affected the survey?	☐	☐	☐	☐	☐

INT4 How tired did the patient appear after the interview?

☐1	Very
☐2	Somewhat
☐3	Not at all
