# Supplementary material for: The prevalence of perceived stigma and self-blame and their associations with depression, emotional well-being and social well-being among advanced cancer patients: evidence from the APPROACH cross-sectional study in Vietnam
Source: BMC Palliat Care. 2021 Jul 7;20:104. doi: 10.1186/s12904-021-00803-5 (PMC8265020; doi:10.1186/s12904-021-00803-5)
Supplement: Supplementary file 2 — Additional file 2. Holm’s correction for the p-values [file 12904_2021_803_MOESM2_ESM.docx]

**Additional File 2. Holm’s correction for the p-values**

In order to control for the Type 1 error inflation, we used corrected p-values using the Holm’s method which is commonly used for this purpose.

The Holm’s correction ranks the p-values of all the comparisons being made. The critical p-value for the *i*th p value (with *i*=1 being the smallest p-value), p_i_ , will then be:

$$\frac{\alpha}{n-i+1}$$

Where *n* is the number of comparison (9 in this study) and α the original critical p-value (0.05 in this study) to be compared against. The comparisons are made as long as the previous p-value is significant, the procedure stops at the first non-significant finding and all previous comparisons will be considered as significant, correspondingly, all comparisons afterwards are considered non-significant.
