# Supplementary material for: The prevalence of perceived stigma and self-blame and their associations with depression, emotional well-being and social well-being among advanced cancer patients: evidence from the APPROACH cross-sectional study in Vietnam
Source: BMC Palliat Care. 2021 Jul 7;20:104. doi: 10.1186/s12904-021-00803-5 (PMC8265020; doi:10.1186/s12904-021-00803-5)
Supplement: Supplementary file 3 — Additional file 3. Full list of estimates [file 12904_2021_803_MOESM3_ESM.docx]

**Additional File 3. Full list of estimates.**

**Additional Table 1. Associations of perceived stigma with depression, emotional well-being and social well-being.**

|  | **Model 1:**  **Depressive symptoms**  **(CES-D)** ^a^ | | **Model 2:**  **Emotional**  **well-being** ^a^ | | **Model 3:**  **Social**  **well-being** ^a^ | |
| --- | --- | --- | --- | --- | --- | --- |
|  | **Coefficient,** ß  **(95% CI)** | **p-value** | **Coefficient,** ß  **(95% CI)** | **p-value** | **Coefficient,** ß  **(95% CI)** | **p-value** |
| **Perceived stigma** | 0.1  (0.1, 0.2) | 0.000 | -0.0 *****  (-0.1, 0.0) | 0.024 | 0.0  (-0.1, 0.0) | 0.098 |
| **Male**  **(ref. female)** | -0.6  (-3.7, 2.5) | 0.687 | -1.3  (-3.2, 0.6) | 0.180 | -0.5  (-2.1, 1.0) | 0.491 |
| **Age** | -0.1  (-0.2, 0.0) | 0.039 | 0.0  (0.0, 0.1) | 0.261 | 0.0  (0.0, 0.1) | 0.183 |
| **Married**  **(ref. not married** | 1.6  (-1.9, 5.2) | 0.355 | -1.7  (-3.9, 0.5) | 0.133 | 2.2  (0.5, 4.0) | 0.013 |
| **Education** | 0.0  (-0.4, 0.3) | 0.806 | 0.0  (-0.3, 0.2) | 0.792 | 0.1  (0.0, 0.3) | 0.119 |
| **Have religious affiliation**  **(ref. no religion)** | 1.6  (-1.9, 5.2) | 0.355 | -0.4  (-2.0, 1.1) | 0.567 | 1.0  (-0.3, 2.2) | 0.122 |
| **Financial distress** | 1.3  (0.6, 1.9) | 0.000 | -0.7  (-1.1, -0.4) | 0.000 | -0.1  (-0.4, 0.2) | 0.614 |
| **Aware of disease severity (ref. not aware)** | 2.0  (-0.5, 4.6) | 0.116 | -2.3  (-3.9, -0.7) | 0.005 | -0.3  (-1.9, 1.3) | 0.703 |
| **Lung cancer** | 4.6  (1.3, 7.8) | 0.006 | -0.7  (-2.7, 1.4) | 0.519 | -0.3  (-1.9, 1.3) | 0.703 |
| **Breast cancer** | 2.7  (-1.1, 6.4) | 0.162 | -2.1  (-4.4, 0.3) | 0.081 | -0.2  (-2.1, 1.7) | 0.834 |
| **Colorectal cancer** | -4.7  (-8.9, -0.5) | 0.030 | -0.7  (-3.3, 1.9) | 0.609 | -0.4  (-2.5, 1.7) | 0.711 |
| **Nasopharyngeal cancer** | -6.2  (-10.4, -1.9) | 0.005 | 3.6  (0.9, 6.3) | 0.008 | 1.4  (-0.7, 3.6) | 0.183 |
| **Constant** | 17.0  (7.6, 26.3) | 0.000 | 19.2  (13.4, 25.0) | 0.000 | 17.4  (12.7, 22.1) | 0.000 |
| **Adj R^2^** | 0.2574 | | 0.1590 | | 0.0604 | |

^a^ Multivariable linear regression controlled for gender, age, marital status, education, religion, financial distress, awareness of disease severity, and type of cancer.

***** denotes statistical significance of the independent variable in question after the Holm’s adjustment.

**Additional Table 2. Associations of behavioural self-blame with depression, emotional well-being and social well-being.**

|  | **Model 4:**  **Depressive symptoms**  **(CES-D)** ^a^ | | **Model 5:**  **Emotional**  **well-being** ^a^ | | **Model 6:**  **Social**  **well-being** ^a^ | |
| --- | --- | --- | --- | --- | --- | --- |
|  | **Coefficient,** ß  **(95% CI)** | **p-value** | **Coefficient,** ß  **(95% CI)** | **p-value** | **Coefficient,** ß  **(95% CI)** | **p-value** |
| **Presence of behavioural self-blame** | 1.7  (-1.0, 4.4) | 0.225 | -1.4  (-3.1, 0.2) | 0.088 | -0.4  (-1.7, 0.9) | 0.538 |
| **Male**  **(ref. female)** | 0.5  (-2.8, 3.8) | 0.757 | -1.3  (-3.3, 0.7) | 0.197 | -0.8  (-2.4, 0.8) | 0.343 |
| **Age** | -0.2  (-0.3, -0.1) | 0.005 | 0.1  (0.0, 0.1) | 0.115 | 0.1  (0.0, 0.1) | 0.089 |
| **Married**  **(ref. not married)** | 1.7  (-2.0, 5.3) | 0.361 | -1.7  (-4.0, 0.5) | 0.121 | 2.2  (0.5, 4.0) | 0.013 |
| **Education** | 0.0  (-0.4, 0.4) | 0.947 | -0.1  (-0.3, 0.2) | 0.672 | 0.1  (0.0, 0.3) | 0.132 |
| **Have religious affiliation**  **(ref. no religion)** | -0.2  (-2.7, 2.3) | 0.881 | -0.6  (-2.2, 0.9) | 0.425 | 0.9  (-0.3, 2.1) | 0.148 |
| **Financial distress** | 1.4  (0.7, 2.0) | 0.000 | -0.8  (-1.2, -0.4) | 0.000 | -0.1  (-0.4, 0.2) | 0.514 |
| **Awareness of disease severity (ref. not aware)** | 1.2  (-1.4, 3.9) | 0.350 | -2.0  (-3.6, -0.4) | 0.016 | -0.7  (-2.0, 0.6) | 0.293 |
| **Lung cancer** | 4.4  (1.0, 7.8) | 0.010 | -0.6  (-2.6, 1.4) | 0.570 | -0.3  (-1.9, 1.4) | 0.739 |
| **Breast cancer** | 4.0  (0.2, 7.9) | 0.038 | -2.5  (-4.8, -0.2) | 0.035 | -0.5  (-2.4, 1.4) | 0.611 |
| **Colorectal cancer** | -5.0  (-9.5, -0.6) | 0.027 | -0.8  (-3.5, 1.9) | 0.558 | -0.2  (-2.4, 1.9) | 0.825 |
| **Nasopharyngeal cancer** | -7.4  (-11.9, -3.0) | 0.001 | 4.3  (1.5, 7.0) | 0.002 | 1.7  (-0.4, 3.9) | 0.117 |
| **Constant** | 20.5  (10.9, 30.1) | 0.000 | 18.4  (12.6, 24.2) | 0.000 | 16.6  (11.9. 21.3) | 0.000 |
| **Adj R^2^** | 0.2061 | | 0.1488 | | 0.0512 | |

^a^ Multivariable linear regression controlled for gender, age, marital status, education, religion, financial distress, awareness of disease severity, and type of cancer.

***** denotes statistical significance of the independent variable in question after the Holm’s adjustment.

**Additional Table 3. Associations of characterological self-blame with depression, emotional well-being and social well-being.**

|  | **Model 7:**  **Depressive symptoms**  **(CES-D)** ^a^ | | **Model 8:**  **Emotional**  **well-being** ^a^ | | **Model 9:**  **Social**  **well-being** ^a^ | |
| --- | --- | --- | --- | --- | --- | --- |
|  | **Coefficient,** ß  **(95% CI)** | **p-value** | **Coefficient,** ß  **(95% CI)** | **p-value** | **Coefficient,** ß  **(95% CI)** | **p-value** |
| **Presence of characterological self-blame** | 3.0 *****  (0.5, 5.5) | 0.020 | -1.6 *****  (-3.1, -0.1) | 0.038 | 0.8  (-0.4, 2.0) | 0.191 |
| **Male**  **(ref. female)** | 1.0  (-2.1, 4.1) | 0.514 | -1.8  (-3.7, 0.1) | 0.064 | -1.0  (-2.5, 0.5) | 0.210 |
| **Age** | -0.2  (-0.3, 0.0) | 0.006 | 0.1  (0.0, 0.1) | 0.137 | 0.1  (0.0, 0.1) | 0.081 |
| **Married**  **(ref. not married)** | 1.5  (-2.1, 5.1) | 0.410 | -1.6  (-3.9, 0.6) | 0.141 | 2.2  (0.4, 4.0) | 0.015 |
| **Education** | -0.1  (-0.5, 0.3) | 0.742 | 0.0  (-0.3, 0.2) | 0.849 | 0.1  (-0.1, 0.3) | 0.177 |
| **Have religious affiliation**  **(ref. no religion)** | -0.3  (-2.8, 2.2) | 0.817 | -0.6  (-2.1, 1.0) | 0.470 | 0.9  (-0.3, 2.1) | 0.156 |
| **Financial distress** | 1.3  (0.6, 1.9) | 0.000 | -0.7  (-1.1, -0.4) | 0.000 | -0.1  (-0.4, 0.2) | 0.501 |
| **Awareness of disease severity (ref. not aware)** | 1.4  (-1.2, 3.9) | 0.340 | -2.1  (-3.6, -0.5) | 0.011 | -0.7  (-2.0, 0.6) | 0.271 |
| **Lung cancer** | 4.7  (1.4, 8.0) | 0.006 | -0.7  (-2.8, 1.3) | 0.471 | -0.2  (-1.8, 1.4) | 0.802 |
| **Breast cancer** | 3.8  (0.1, 7.6) | 0.047 | -2.4  (-4.7, -0.1) | 0.042 | -0.6  (-2.4, 1.3) | 0.544 |
| **Colorectal cancer** | -5.5  (-9.9, -1.1) | 0.016 | -0.6  (-3.3, 2.1) | 0.677 | -0.4  (-2.5, 1.8) | 0.736 |
| **Nasopharyngeal cancer** | -6.7  (-11.1, -2.4) | 0.003 | 3.7  (1.1, 6.4) | 0.006 | 1.7  (-0.5, 3.8) | 0.122 |
| **Constant** | 19.7  (10.2, 29.2) | 0.000 | 18.6  (12.9, 24.4) | 0.000 | 16.2  (11.6, 20.9) | 0.000 |
| **Adj R^2^** | 0.2229 | | 0.1552 | | 0.1488 | |

^a^ Multivariable linear regression controlled for gender, age, marital status, education, religion, financial distress, awareness of disease severity, and type of cancer.

***** denotes statistical significance of the independent variable in question after the Holm’s adjustment.
